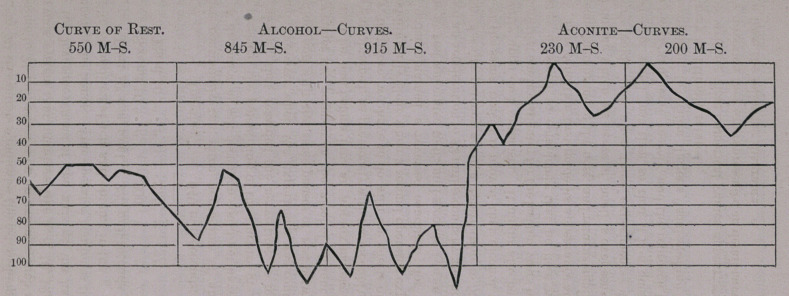# A New Discovery in the Service of Homœopathy

**Published:** 1881-12

**Authors:** Manuel Græter


### A NEW DISCOVERY IN THE SERVICE OF HOMŒOPATHY.

*Translated from " Populare Zeitschrift fur Homœopathie," Jan.* 1881.

(*Continued from page 546.*)

By Prof. Manuel Græter.

With the same operator whose nerve-time on an average amounted to 55 mill-seconds, the following results following the inhaling of alcohol were found. Position of the hands 0.

| | | | | |
|---|---|---|---|---|
| 1st act | 75 | —— | difference, | 75 mill-seconds. |
| 2nd " | 165 | —— | " | 90 " " |
| 3rd " | 220 | —— | " | 55 " " |
| 4th " | 280 | —— | " | 60 " " |
| 5th " | 360 | —— | " | 80 " " |
| 6th " | 465 | —— | " | 105 " " |
| 7th " | 560 | —— | " | 95 " " |
| 8th " | 635 | —— | " | 75 " " |
| 9th " | 735 | —— | " | 100 " " |
| 10th " | 845 | —— | " | 110 " " |
| 11th " | 935 | —— | " | 90 " " |
| 12th " | 1040 | —— | " | 105 " " |
| 13th " | 1105 | —— | " | 65 " " |
| 14th " | 1185 | —— | " | 80 " " |
| 15th " | 1270 | —— | " | 85 " " |
| 16th " | 1370 | —— | " | 100 " " |
| 17th " | 1475 | —— | " | 105 " " |
| 18th " | 1565 | —— | " | 90 " " |
| 19th " | 1650 | —— | " | 85 " " |
| 20th " | 1760 | —— | " | 110 " " |
| | 1760 | —— | " | 1760 mill-seconds. |

These figures, therefore, show a retardation of nerve-time; for divided by 20, we find it descended to 88 mill-seconds, hence a difference of 33, as compared with the state of rest. Let us remark that our operator collected his whole energy and strength of will, in order to work as evenly as at other times at the apparatus. That he did not succeed we see by the figures. Evidently there appeared a slight intoxication by the inhaling of the alcohol, for his head was benumbed and some giddiness existed. After a lapse of 15 minutes, during which he had breathed fresh air out of doors, the experiment is repeated *without* inhaling alcohol and furnishes the following results:

| | | | | |
|---|---|---|---|---|
| 10 | acts | 600 | mill-seconds. | |
| 10 | " | 610 | " | " |
| 10 | " | 580 | " | " |
| 10 | " | 595 | " | " |
| 10 | " | 560 | " | " |

The nerve-time amounts therefore on an average, when that number is divided by 50, to 58 mill-seconds: it approaches the former figure-of-rest, differing merely by 3 mill-seconds from it.

Hereupon the experiment with the medicine is made, by inhaling a 15th potency of *Aconitum Napellus* prepared with the same alcohol, from a glass vessel for the space of two minutes and setting the instrument going again. Evidently the same alcohol intoxication as before ought to have appeared, similar figures as with the inhaling of alcohol ought to have been obtained. But this is by no means the case, for in this experiment we obtained the following figures:

State of hands 0.

| | | | | | | |
|---|---|---|---|---|---|---|
| 1st act | 45 | —— | difference, | 45 | mill-seconds. | |
| 2nd " | 75 | —— | " | 30 | " | " |
| 3rd " | 115 | —— | " | 40 | " | " |
| 4th " | 140 | —— | " | 25 | " | " |
| 5th " | 160 | —— | " | 20 | " | " |
| 6th " | 165 | —— | " | 5 | " | " |
| 7th " | 173 | —— | " | 8 | " | " |
| 8th " | 185 | —— | " | 12 | " | " |
| 9th " | 205 | —— | " | 20 | " | " |
| 10th " | 230 | —— | " | 25 | " | " |
| 11th " | 245 | —— | " | 15 | " | " |
| 12th " | 0 | —— | " | ... | " | " |
| 13th " | 255 | —— | " | 10 | " | " |
| 14th " | 273 | —— | " | 18 | " | " |
| 15th " | 295 | —— | " | 22 | " | " |
| 16th " | 320 | —— | " | 25 | " | " |
| 17th " | 350 | —— | " | 30 | " | " |
| 18th " | 385 | —— | " | 35 | " | " |
| 19th " | 410 | —— | " | 25 | " | " |
| 20th " | 430 | —— | " | 20 | " | " |
| | | | | 430 | mill-seconds. | |

Divided by 20 this number indicates the astonishing shortening of the nerve-time to 21 mill-seconds on an average; such an excitement of the nervous system that numbers like 5, 8, 10, 12, appear; and once only the lever-movement of the axis of the hands occurred, without moving the hands. Our operator continues the inhaling of *Aconite* 15 in 30 additional acts and obtains in the

| | | |
|---|---|---|
| First 10 acts, total, | 225 | mill-seconds. |
| Second 10 " " | 200 | " " |
| Third 10 " " | 215 | " " |
| Together, | 640 | mill-seconds. |

Therefore continually the same nerve-time of about 21 mill-seconds. A deception or self-deception could not have taken place; for in our presence the potentizing of the tincture of *Aconite;* with the same alcohol that retarded the nerve-time so much, was performed, and moreover when the experiment was continued, in the same position and with the same pauses of rest between; inhaling alternately either the potency or the alcohol without the operator's knowing what he had before him, an approximately similar group of figures was obtained as previously with the alcohol or the potency. Decidedly, therefore, we have to do with the effects of *Aconite,* this becomes additionally clear when a second operator experimenting in the same order obtained a reversed condition; for with him the nerve-time at rest amounted to 62 mill-seconds (on an average), and experienced an acceleration to 42 mill-seconds by alcoholic inhalation, and by the *Aconite* potency again a retardation to 52 mill-seconds, by which at the same time it is proved that alcohol and medicines act differently upon each organism. It might also be of interest to mention here that one investigator for the last ten years has temporarily lost the sense of smell so completely that he does not even smell camphor. This condition first made its appearance after diphtheria of the mucous membrane of the nose, and returns again when a running cold during which his nerve of smell is over-sensitive, changes into a dry cold, and when these experiments were made he had no sense of smell just then, so that it is a question of direct effects on blood and nerve merely, and the reproach that we homœopathists endeavor to utilize Jæger's "soul-*smelling*" for scientific purposes may be fittingly refuted.

But how striking these numerical values appear, when according to Jæger's direction they are represented in detail-curves, the reader may learn from the following diagram:

CURVE OF REST.
550 M-S.
ALCOHOL—CURVES.
845 M-S.
915 M-S.
ACONITE—CURVES.
230 M-S.
200 M-S.
10
20
30
40
50
60
70
80
90
100

Now for Prof. Jæger's indicated purposes, a few such detail curves do not suffice, but first, after the operator is bodily and mentally composed and has manipulated the apparatus breathing freely, ten acts (one decade) without inhalation, then 90 acts with inhaling of alcohol are performed. These 100 acts constitute the first half of an osmogram reduced to 10 decade figures. Upon this 100 acts, (10 decades) under the influence of inhaling a potency follow. From the figures thus obtained the difference between rest and alcohol and between the latter and the homoeopathic medicinal potency prepared with it, is calculated in percentage. Upon this calculation rest Prof. Jæger's statements: that certain medicaments in certain potencies produce a difference of 40 or 50 per cent. as contrasted with pure alcohol. Each single average value of a decade is besides graphically represented in Dr. Jæger's book. As these diagrams are colored green and red we cannot reproduce them.

Finally Prof. Jæger has not made all the examinations of homœopathic medicaments in his own person, but the greater part of those communicated by him were undertaken by three students (Gohrum, Pantzer and Schlichter) who worked under his direction. This places the discoverer of these valuable facts beyond the reproach made from hostile quarters: *that all that he maintained was only a spectre of his imagination or a hallucination.* These gentlemen investigated the following medicaments:

*Aconitum.* This medicament in its mother tincture caused a retardation of the nerve-time of 14.7 per cent. with Prof. Jæger; in its potencies however an essential increase of excitability and an acceleration of nerve-time viz: in its 5th potency, 10.6%; 10th potency, 40 per cent.; 15th, 47.5 per cent.; 20th, 39 per cent.; 30th, 25.3 per cent.; 100th, 29.3 per cent.; 150th, 35.2 per cent. With Mr. Gohrum, tincture of *Aconite* produced an acceleration of 25 per cent. The 1st to 10th potencies did not accelerate it so much as the tincture; on the other hand the 15th potency caused 39 per cent. just as with Jæger. Then the heightening of excitability was again decreased with the succeeding potencies, until with the 200th potency, there appears again a maximum of 36 per cent. With Mr. Schlichter the maximum coincided likewise with the 15th potency, and a new maximum made its appearance again only with the 100th.

We can therefore in employing *Aconite,* rest satisfied with the 15th potency and need not resort to higher.

*Thuja.* This medicament was tested almost exclusively by Mr.

Gohrum in the 1st and 1000th potency. Here also the maximum of excitability coincides with the 15th potency with 70.6 per cent. Yet already the 1st potency produced 40.7 per cent. From the 15th potency onward the effects become weaker and stronger only in a few potencies, without however attaining this maximum; for we find with the 30th potency, 61.6 per cent.; with the 300th, 67.6 per cent.; with the 400th, 42 per cent.; with the 100th, 63.6 per cent. This is in accord with our own experience. With us too the maximum with 62 per cent. was in the 15th potency; an increase of excitability of 57 per cent. already in the 3d potency, and the 30th produced 28 per cent. only.

*Natrum Muriaticum*, tested from 2d to 2000th potency by Prof. Jæger and Messrs. Gohrum and Pantzer. The 2d potency caused with Prof. Jæger an increase of excitability of 10 per cent.; the 10th, 19 per cent.; the 15th, 38 per cent.; the 30th, 25.8 per cent.; the 100th, 25 per cent.; the 200th, 43 per cent.; the 500th, 47.5 per cent.; the 1000th, 28.8 per cent.; the 2000th, measured for greater security's sake three times, 60, 56 and 55.3 per cent. Therefore here also a first maximum is situated in the 15th potency, the 2d in the 200th; the 3d in the 500th; the 4th in the 2000th. The 200th and 1000th show weaker effects than the 15th.

Since it is shown by Spectrum Analysis that the atmosphere everywhere contains atoms of *Natrum Muriaticum*, it would appear at first sight that the heightening of the effects was not owing to the medical substance employed for potentizing, but that the alcohol whilst being shaken in the flask absorbed atoms of this substance from the air. A potentizing of pure alcohol up to 100 was therefore performed, consequently shaken up with air. The curves obtained with the alcohol thus shaken showed the error of this surmise, for between the alcohol-figure and that of the potentized alcohol there was a difference of only 7 per cent.

That, for the rest, in this medicament idiosyncrasy performs a quite particular part, may be seen from the fact that the author of this article who uses this remedy with predilection wherever it is suitable and can record quite extraordinary results with patients, could not make any impression upon himself during his neural analytical experiments. The results obtained on different days in different experimental series differed but by few per cent. from the effects of alcohol.

Mr. Gohrum on the contrary obtained quite considerable effects from *Natrum Muriaticum*. For the 10th potency produced with him

71.6 per cent.; the 20th, 72 per cent.; the 30th, 20.6 per cent.; the 500th, 74.5 per cent.; the 2000th, 73.6 per cent. This may be owing to the fact that Mr. Gohrum possesses a highly developed sense of smell, and states in the most positive manner that he is able to distinguish this medicament in the 10th, 20th, and higher potencies from alcohol, without having recourse to the apparatus. Similar observations were likewise made by some other investigators with acute sense of smell.

*Aurum.* In the tests made by Mr. Schlichter the 5th potency produced 1.1 per cent.; the 10th, 9.4 per cent.; the 15, 18.2 per cent.; the 20th, 12 per cent.; the 30th, 21.4 per cent.; the 100th, 29.1 per cent.; the 200th, 37.9 per cent.; the 400th, 30.4 per cent.; the 500th, 32.9 per cent.

These figures of course have but an abstract value, for in an experiment made on another day with the same potency a greater or less difference may result from causes mentioned at an earlier stage. But a potency will always be distinguished in its effect from that alcohol with which it was prepared. Let that suffice us. We must refrain from further entering into Prof. Jæger's given explanation of this discovery which indeed almost approaches the miraculous, and which enlarges the former conceptions of the divisibility of matter and the effects of this matter diluted to infinity upon the human body—just as much as the telescope enlarges our conception of the magnitude of the universe.

We must reserve for future occasions the details of experiments made and still to be made by us with other homœopathic medicaments, for they would not be suitable for this popular account. We limit ourself here to pointing out that by this discovery many things were confirmed that till now were theoretically supposed or deduced from practical experience. It refutes the assumption that extremely high potencies are ineffective. On the other hand the heightening of the effects which appeared with all operators up to the 15th potency and then the gradual weakening of the same to the 30th, does not at all justify the advocates of high potencies in looking down with contempt upon the adherents of low dilutions. Do we not, for instance, find an extraordinary heightening of excitability with Mr. Gohrum (so very sensitive to high potencies) even with the 1st potency of *Thuja*, and the strongest increase with the 15th? But if we know that idiosyncrasy with all patients plays so great a part in the results, and that different individuals are so differently affected by different medical substances, as the operators by the neural-analytical apparatus, we may in spite of the very gratifying results which Jæger's experiments in reference to high potencies afford, maintain now as before that he who through predilection treats his patients exclusively with the 1000th, 200th, or 30th potency, is not to be called a faithful disciple of homœopathy, but that *that physician is at all events the best who for use in his practice has the entire scale of potencies at his disposal.*

To Prof. Jæger and his pupils we sincerely believe ourselves to be most sincerely indebted for their services to the homœopathic curative method. But to the adversaries of our cause who probably will endeavor to dispose of this discovery with theoretical reasonings or with defamations of Prof. Jæger's person, we will now exclaim in Hahnemann's words: "*Imitate it, but imitate it exactly.*"

## Figures and Tables

**Figure f1:**